# Bioactive Natural Products in Cardiovascular Disease: Focus on Thrombotic Events

**DOI:** 10.1002/ptr.70415

**Published:** 2026-06-29

**Authors:** Maria Nektaria Magkoulia, Despoina Pantazi

**Affiliations:** ^1^ Laboratory of Biochemistry, Department of Chemistry University of Ioannina Ioannina Greece

**Keywords:** cardiovascular disease, natural products, neutrophil extracellular traps, thrombosis

## Abstract

Bioactive compounds derived from natural products (NPs) continue to attract considerable scientific interest due to their wide range of beneficial biological activities. This review examines the role of these compounds in the prevention and management of cardiovascular disease (CVD), with particular emphasis on their involvement in thrombotic processes. Specifically, this review addresses whether bioactive NPs can serve as complementary therapeutic agents in preventing and modulating thrombotic events associated with CVD. Special attention is given to natural food sources with well‐documented antithrombotic properties, as well as to current methodologies used for the processing, extraction, and isolation of their biologically active constituents. Furthermore, emerging evidence regarding the impact of chronic dietary intake of bioactive compounds on the pharmacokinetics and therapeutic efficacy of antithrombotic agents is discussed, including potential interactions with commonly prescribed anticoagulant drugs. The contribution of nutritional epigenetics to personalized strategies for CVD prevention and treatment—especially through gene–diet interactions—is also explored. In addition, the growing role of artificial intelligence in accelerating the discovery and optimization of novel natural antithrombotic agents is highlighted within the context of recent technological advances. Overall, this review underscores the potential of NPs as integral components of innovative and complementary strategies for CVD management, primarily through their pronounced antiplatelet and antithrombotic activities.

## Graphical abstract

An overview of the role of natural products (NP) in cardiovascular disease prevention, highlighting key mechanisms of action: including antiplatelet, antithrombotic, anti‐inflammatory, and antioxidant effects. Additionally, it illustrates the integration of diet–drug interactions, nutritional epigenetics, and the role of artificial intelligence in advancing personalized cardiovascular prevention strategies.

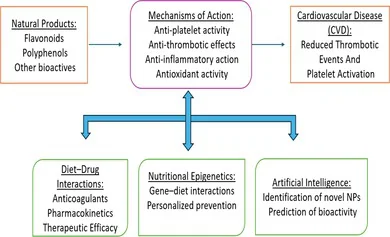

**Abbreviations**
ADA: adenosine deaminase
ADP: adenosine diphosphate
AI: artificial intelligence
ASA: acetylsalicylic acid
AT1R: angiotensin II Type 1 receptor
Ca^2+^
: cytosolic calcium
CAMP: cyclic CMP
CASP: computer‐aided synthesis planning
COX: cyclooxygenase
CYP: cytochrome P450
DDIs: drug–drug interactions
DNA: deoxyribonucleic acid
DNMTs: DNA methyltransferases
DPPH: 2,2‐diphenyl‐1‐picrylhydrazyl
EGCG: epigallocatechin gallate
Exog‐miRNA: exogenous miRNA
FRAP: ferric reducing antioxidant power
GPIIb/IIIa: integrin αIIbβ3
HDAC: histone deacetylase
ICAM‐1: intercellular adhesion molecule‐1
LDL: low‐density lipoprotein
Lyso‐PAF AT: lyso‐PAF acetyltransferase
MD: mediterranean diet
MiRNA: microRNA
ML: machine learning
MPO: myeloperoxidase
NADPH: nicotinamide adenine dinucleotide phosphate (reduced form)
NET: neutrophil extracellular traps
NFs2: nuclear factor erythroid 2–related factor 2
NO: nitric oxide
NP: natural product
PAD4: arginine deiminase 4
PAF: platelet activating factor
PDI: protein disulfide isomerase
PF4: platelet factor 4
PGE2: prostaglandin E2
PGH2: prostaglandin H2
PGI2: prostacyclin
PMQ: pentamethyl quercetin
ROS: reactive oxygen species
RYR: red yeast rice
TCHM: traditional Chinese herbal medicinal product
TF: tissue factor
TXA2: thromboxane A2
vWF: von Willebrand Factor

## 1 Introduction

Natural products (NPs) have historically played a key role in drug discovery because of their diverse properties, such as high scaffold diversity, high structural complexity, high molecular weight, a high number of sp^3^ carbon atoms, combined with fewer nitrogen and halogen atoms, and a high number of H‐bond acceptors and ligands (Atanasov et al. 2021).

Bioactive compounds from NPs are isolated and analyzed to identify potential treatments for chronic conditions such as thrombosis (Mazumder et al. 2022). Plants and herbs contain a variety of compounds such as bioactive metabolites, potent antioxidants, and anti‐inflammatory compounds, which can inhibit multiple enzymes and thereby exhibit important anticoagulant and antiplatelet properties (Lamponi 2021).

Many NPs derived from plants, animals, and microorganisms demonstrate antithrombotic potential (Ohkura and Morimoto‐Kamata 2024). Notable examples are hirudin, a thrombin‐specific inhibitor derived from *Hirudo medicinalis* , and isoquercitrin, the first natural inhibitor of protein disulfide isomerase (PDI). These compounds are currently being studied in clinical trials. These results demonstrate the potential of NPs as templates for developing new antithrombotic drugs (Yuan et al. 2024).

The reasons for using NPs as an alternative or complement to conventional antithrombotic drugs, in addition to their safety, include lower costs compared to synthetic drugs, the presence of multiple active components, high therapeutic efficacy and, most importantly, fewer adverse effects on the human body (Chen et al. 2015).

### 1.1 Bioactive Ingredients of NPs

The medicinal properties of plants have been recognized since antiquity, and their use for therapeutic and preventive purposes has been widespread (Lamponi 2021) through the consumption of herbal products (Mutha et al. 2021). Modern research has increasingly focused on bioactive molecules extracted from plants and their potential therapeutic applications which appear to depend on the nature and diversity number of the chemical compounds present (Lamponi 2021).

### 1.2 Primary and Secondary Metabolites

Plant‐derived molecules are classified into primary and secondary metabolites according to their role in growth and development. Primary metabolites are compounds essential for the metabolism and growth of plants and therefore produced in relatively high amounts as they are continuously required by the plants. These metabolites generally do not exhibit direct pharmacological activity (Lamponi 2021).

Secondary metabolites are organic molecules that are not directly involved in the growth or development of plants and, therefore, their absence has no significant impact on plant survival. However, they play a key role in interspecies interactions, including defense mechanisms (Lamponi 2021). Secondary metabolites possess high therapeutic potential, especially in classes such as polyphenols and flavonols. Long‐term dietary intake of these compounds seems to produce protective effects and may even prevent the onset of cardiovascular disease, diabetes, and some types of cancers (Mutha et al. 2021).

### 1.3 Phenolic Compounds, Polyphenols, Flavonoids

Phenolic compounds include polyphenols and flavonoids and constitute the main group of secondary metabolites widely distributed in plants and their extracts. These compounds have potent antioxidant, anti‐inflammatory, antiplatelet, and anticoagulant properties; therefore, polyphenol‐rich plant extracts can be a valuable therapeutic agent in the prevention and treatment of thromboembolic complications (Lamponi 2021).

Polyphenols are one of the most important groups of naturally occurring secondary metabolites and are characterized by the presence of at least one phenolic ring. They are abundant in vegetables, fruits, cereals, and nuts (Mutha et al. 2021). In plants, polyphenols are usually present as glycosides, in which the phenolic structure is conjugated with the sugar's moieties (Lamponi 2021). Structurally, these compounds may contain different phenolic units linked to organic acids and carbohydrates (Mutha et al. 2021).

Phenolic compounds are generally classified into flavonoids and nonflavonoids (Lamponi 2021) (Figure 1). Flavonoids are a large subgroup of polyphenols and are generally recognized as phytochemicals with relatively low molecular weights (Mutha et al. 2021; Jia et al. 2021; Bojić et al. 2019). These flavoring compounds contribute to the characteristic color and aroma of plants and belong to a very heterogeneous group of NPs (Mutha et al. 2021; Bojić et al. 2019).

**FIGURE 1:**
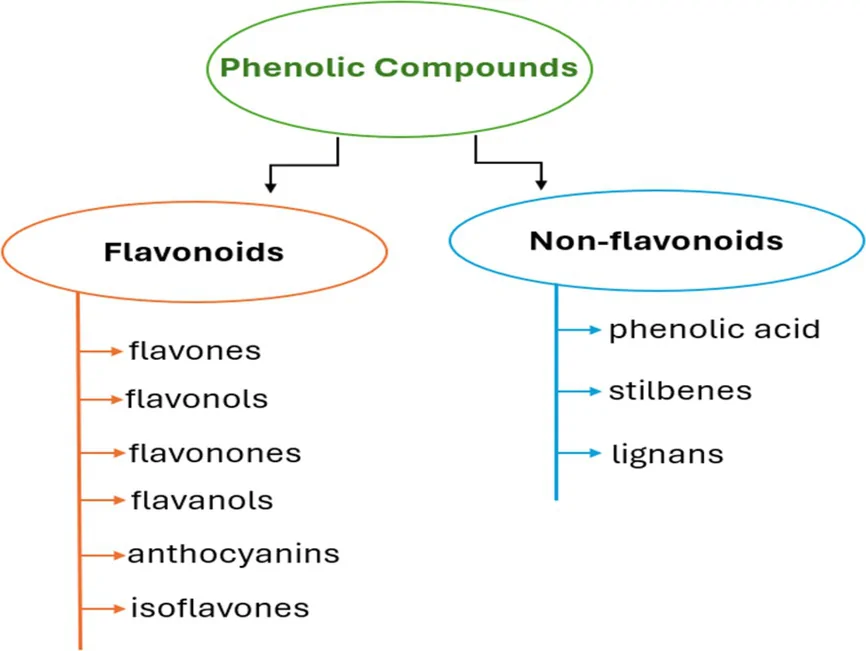
Classification of phenolic compounds into flavonoids and nonflavonoids, highlighting the main subclasses of flavonoids and nonflavonoids.

Flavonoids are classified into different subclasses according to different criteria, such as their chemical structure, degree of unsaturation, and oxidation state of the heterocyclic ring (Jia et al. 2021; Bojić et al. 2019). Their structural diversity is illustrated in Figure 2.

**FIGURE 2:**
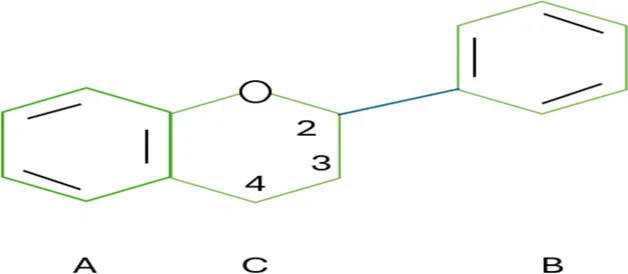
Structural characteristics of flavonoids.

Major subclasses include flavones, flavonols, flavanones, isoflavones, and anthocyanins, as summarized in Figure 3.

**FIGURE 3:**
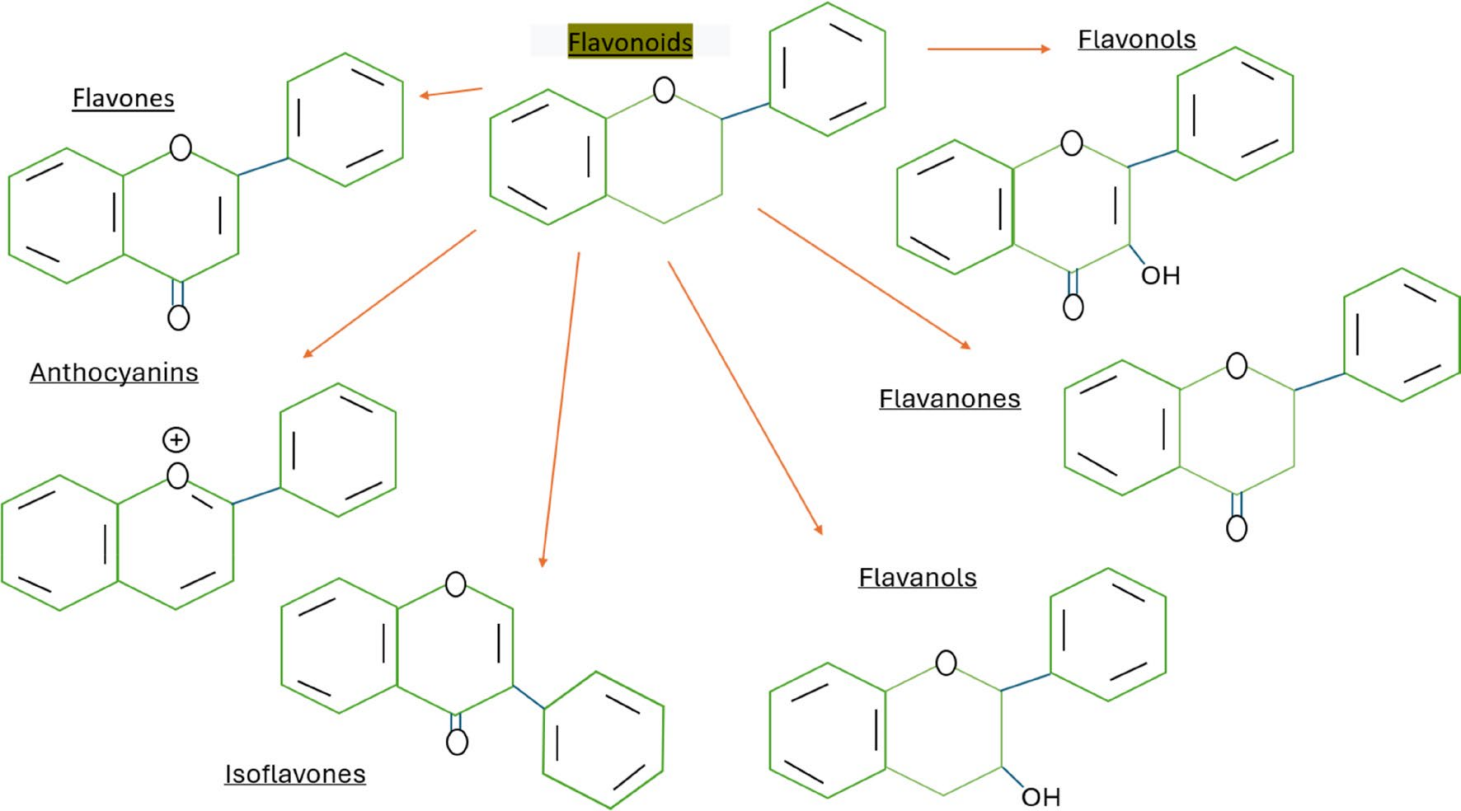
Schematic representation of the basic structure of flavonoids (C6–C3–C6) and their classification into specific subcategories based on structural characteristics.

## 2 Properties of Natural Products

NPs have a wide range of biological activities, including anticancer, neuroprotective, hepatoprotective, antiviral, fungicidal, and osteoporotic benefits (Bojić et al. 2019). However, their most notable activity is their anti‐inflammatory and antioxidant properties, and antithrombotic and antiplatelet properties that will be specifically addressed in this article (Table 1).

**TABLE 1:** Food substances with characteristic antithrombotic activity.

Substance	Source of origin	Source of origin antithrombotic activity	References
Adenosine and other nucleosides (adenosine and other nucleosides)	Tomatoes ( *Solanum lycopersicum* ), Fresh and processed tomatoes	Platelet inhibition occurs through interaction with the ADP receptors P2Y1 and P2Y12	Vilahur and Badimon (2013)
Nucleotides, nucleosides, simple phenolic derivatives, and flavonoid derivatives	Water‐soluble tomato extract (fruitflow, water soluble tomato extract)	– Inhibition of platelet aggregation by reducing intracellular Ca^+2^ mobilization and increased formation of cyclic CMP (cAMP) – Reduction of platelet reactivity and cardioprotection through inhibition of angiotensin‐converting enzyme and relaxation of vascular endothelium	Vilahur and Badimon (2013); Duttaroy (2024); O'Kennedy et al. (2021); O'Kennedy, Raederstorff, et al. (2017)
Monacolin K or lovastatin	Red yeast rice	Inhibition of cholesterol synthesis, reduction of atherosclerotic plaque formation and improvement of endothelial function	Zhu, Qi, et al. (2019)
Phenolic compounds hydroxytyrosol, oleuropein	Olive oil	Reduction of platelet aggregation induced by collagen, arachidonic acid, ADP. Limitation of thromboxane A2 (TXA2) synthesis	Vilahur and Badimon (2013)
Allicin and ajoene, self‐condensation product of allicin	Garlic (*Allum sativum*)	Inhibition of GPIIb/IIIa receptor functionality on platelet membrane hence inhibition of fibrinogen and vWF binding to the receptor	Vilahur and Badimon (2013)
Reseveratrol, the most common compound of stilbenes = phenolic compounds in wine, present in grape skin and seeds (resveratrol, found in grape skin and seeds)	Wine	Reduction of platelet aggregation in a concentration‐dependent manner induced by thrombin, collagen, ADP associated with an increase in cAMP—decrease in Ca^+2^ Antiplatelet action is also due to inhibition of accumulation induced by platelet activating Factor (PAF)	Vilahur and Badimon (2013); Choleva et al. (2025)
Catethin, epicatechin, quercetin	Winery byproducts—aqueous ethanol GP extracts (extracts of winery byproducts—aqueous ethanol GP extracts)	Inhibition of platelet aggregation derived from thromboxane A2 (TXA2), thrombin, ADP	Choleva et al. (2025)
Quercetin, epicatechin, procyanidin, cocoa‐red = the color component of cocoa = the color component of cocoa	Cocoa and dark chocolate (cocoa and dark chocolate)	Reduction of platelet aggregation through reduction of ADP activation of the GPIIb/IIIa receptor and P‐selectin	Vilahur and Badimon (2013); Pearson et al. (2002)
Lignan and phenol	Myristica fragnans (*Myristicaceae*) commonly known as nutmeg	Inhibition of the fibrinolytic pathway activated by urokinase	Sawetaji and Aggarwal (2022)
Gingerols and sogaols, phenolic compounds	Ginger ( *Zingiber officinale* Roscoe)	It restricts the activity of platelet adenosine deaminase (ADA) and increases adenosine levelels, which prevents platelet aggregation	Mao et al. (2019)
Curcumin	Turmeric	Protection against atherosclerosis by enhancing endothelial function and downregulation of angiotensin II type 1 receptor (AT1R) expression	Sharifi‐Rad et al. (2020)
Cinnamophilin is one of the important lignans	Cinnamon *Cinnamomum zeylanicum*, and *Cinnamon cassia*	Blocks thromboxane A2 (TXA2) receptor and causes inhibition of thromboxane synthase and TXA2 receptor antagonist	Rao and Gan (2014)
Berry anthocyanins, cyanidin, pelargonidin, delphinidin, malvidin, peonidin, petunidin	Berries	Improve blood flow, reduce platelet aggregation, and mitigate endothelial cell damage	Golovinskaia and Wang (2021)
Crocin	Saffron— *Crocus sativus*	Effective inhibition of oxidative stress and platelet apoptosis induced by sesamol	Thushara et al. (2014)
Mangiferin	Mango seed extract—*Mangifera Indica L*.	Inhibition of platelet aggregation induced by ADP at 72%	Alañón et al. (2019)
Bromelain (group of proteolytic enzymes)	Pineapple ( *Ananas comosus* )	Prevents the synthesis of blood clotting proteins such as fibrin, by limiting the production of fibrin by reducing some of the intermediates in the clot formation process (such as factor X and prothrombin)	Varilla et al. (2021)
Catechins (GTC) and gallic‐epigallocatechin (EGCG)	Green tea	Inhibited platelet aggregation induced by adenosine diphosphate (ADP) and collagen in the rat ex vivo studies	Kang et al. (1999)
Unsaturated fatty acids such as oleic, linolenic gadoleic, and erucic	Baru almond oil (*Dipteryx alata Vog*.)	Reduce platelet aggregation, reduce production of reactive oxygen species (ROS), and improve vascular function	Silva‐Luis et al. (2022)
Sulforaphane (SFN), a natural isothiocyanate	Cruciferous vegetables such as broccoli and cabbage	Inhibits platelet aggregation through inhibition of thrombin and FXa activity, inhibition of thrombin and FXa production, and reduction of PAI‐1 to t‐PA ratio	Ku and Bae (2014)
Betalains and flavonoids	Beetroot juice rich in nitrates	Reduction of platelet‐monocyte aggregates, which is considered reliable biomarkers of platelet activation	Raubenheimer et al. (2017)
Polyphenols: vitamin C, E, folic acid, anthocyanidins, and flavonols	Green Kiwi *Actinidia deliciosa* —Aqueous extract of kiwi (KFE), green kiwifruit	Inhibition of ADP‐ and collagen‐induced platelet aggregation. While probably not affecting the thromboxane A2 (TXA2) pathway	Duttaroy (2024)

### 2.1 Anti‐Inflammatory Properties

Many compounds derived from NPs also exert potent anti‐inflammatory properties. The precise mechanisms by which NPs exert their anti‐inflammatory effects are not fully understood. Flavonoids may modulate multiple molecular pathways, and individual compounds may act in different ways (Al‐Khayri et al. 2022). Flavonoids are generally thought to reduce inflammation by inhibiting the synthesis of proinflammatory mediators, such as IL‐6, IL‐8, TNF‐α, IL‐1β, and IL‐1β, as well as nitric oxide (NO) and prostaglandin E2 (PGE2) (Bojić et al. 2019).

The anti‐inflammatory properties of flavonoids are closely linked to their chemical structure. Their high antioxidant activity is attributed to the presence of adjacent hydroxyl groups on the B ring (Figure 2). In particular, the structure of the planar ring with C2–C3 double bonds and the position of the hydroxyl groups play a key role in this property. The absence of hydroxyl groups at Positions 3 and 4 of the B ring is associated with a loss of anti‐inflammatory potential (Duttaroy 2024).

### 2.2 Antioxidant Properties

NPs have also gained increasing attention because of their antioxidant properties. Their structural features enhance their ability to scavenge free radicals and chelate metal ions. In particular, excess iron may stimulate the formation of reactive oxygen species (ROS) via the Fenton reaction (Figure 4), which may result in oxidative damage to the cell membrane. Flavonoids may bind iron and form stable complexes, thereby reducing oxidative stress induced by iron (Bojić et al. 2019). In addition, flavonoids and other polyphenols may interact with free radicals derived from lipids and oxygen to disrupt lipid peroxidation chain reactions. Consequently, polyphenols are widely recognized as natural antioxidants that can reduce oxidative damage and inhibit the peroxidation of low‐density lipoproteins (Mutha et al. 2021; Bojić et al. 2019). Flavonoids may also be localized at the lipid‐water interface of the biomolecular framework, where they exert a protective effect against lipid peroxidation. Furthermore, inhibition of key oxidative enzymes such as xanthine oxidase and NADPH oxidase further supports the significant antioxidant potential of these NPs (Bojić et al. 2019).

**FIGURE 4:**
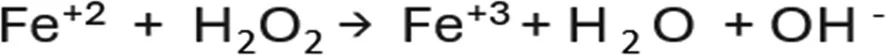
Fenton reaction.

Antioxidant activity is commonly assessed using two main approaches. The FRAP assay (ferric reducing antioxidant power) is used to estimate the total antioxidant capacity based on the reduction of ferric (Fe^3+^) ions to ferrous (Fe^2+^) ions, whereas the DPPH radical scavenging assay evaluates the ability of compounds to neutralize the stable free radical 2,2‐diphenyl‐1‐picrylhydrazyl (O'Kennedy et al. 2021). Additional methods that may be employed include the ABTS radical cation decolorization assay and the Fe^2+^–phenanthroline complex formation assay, which also assess reducing or radical‐scavenging capacity (O'Kennedy, Raederstorff, et al. 2017; Bramki et al. 2025). It is generally recommended to apply at least two different antioxidant assays to obtain a more comprehensive and reliable evaluation of antioxidant activity (O'Kennedy et al. 2021; Ibadullayeva, Kasela, Kozhanova, et al. 2024).

### 2.3 Antidiabetic Properties

Research on Mediterranean edible plants has demonstrated in vitro antidiabetic activity, mainly by the inhibition of enzymes involved in the development of diabetes mellitus. Approximately 85% of these plants were found to inhibit alpha‐glucosidase and alpha‐amylase, enzymes that regulate glucose uptake. Moreover, some studies suggest that Mediterranean plants may stimulate the proliferation of beta‐cells in the pancreas (Zhu, Qi, et al. 2019; Goulas, Banegas‐Luna, Constantinou, Pérez‐Sánchez and Barbout 2022). Specific Mediterranean‐derived foods such as coriander, black mustard, thyme, cumin, strawberries, and olives have been associated with antidiabetic effects in vitro and in vivo. Among the bioactive compounds, flavonoids seem, particularly, promising. Their antidiabetic activity may be mediated by a number of mechanisms, including regulation of insulin secretion and sensitivity, glucose metabolism modulation, and regulation of lipid metabolism. Flavonoids have also been shown to have a high molecular similarity to some antidiabetic medicinal products, which points to their potential—though not yet confirmed—as a natural source of therapeutic compounds. However, further studies are needed to elucidate the structural characteristics of these compounds that contribute to their antidiabetic activity (Zhu, Qi, et al. 2019).

### 2.4 Antithrombotic and Antiplatelet Properties

Flavonoids have shown promising anticoagulant activity in several studies (Vilahur and Badimon 2013; Choleva et al. 2025; Pearson et al. 2002). The first extensive studies carried out in the 20th century indicated that the concentrations needed to obtain an anticoagulant effect in vivo were extremely high when tested in vitro. Later, in 1998, scientists concluded that the anticoagulant properties of flavonoids in vitro are unlikely to be clinically relevant. However, with the development of analytical methods such as thromboelastography and synthesis analysis of resistance, research on flavonoids' antiplatelet and anticoagulant activity has been renewed (Vilahur and Badimon 2013).

The antiplatelet effect of flavonoids, defined as their ability to inhibit the aggregation of platelets thereby preventing thrombus formation, is well‐documented (Vilahur and Badimon 2013). Recent evidence suggests that the major contributor to this activity is flavonoid metabolites produced by the human gut microbiota. Gut bacteria metabolize parent flavonoids to smaller compounds with low bioavailability, leading to increased plasma concentrations of active metabolites (Pearson et al. 2002). Platelets normally act to induce coagulation and stop bleeding; however, overactivation of these cells in pathological conditions may result in excessive aggregation and thromboembolism, which is a major cause of cardiovascular disease and cerebrovascular disorders (Jia et al. 2021). The principal pathways and molecular targets mediating the antiplatelet activity of NPs are illustrated in Figure 5.

**FIGURE 5:**
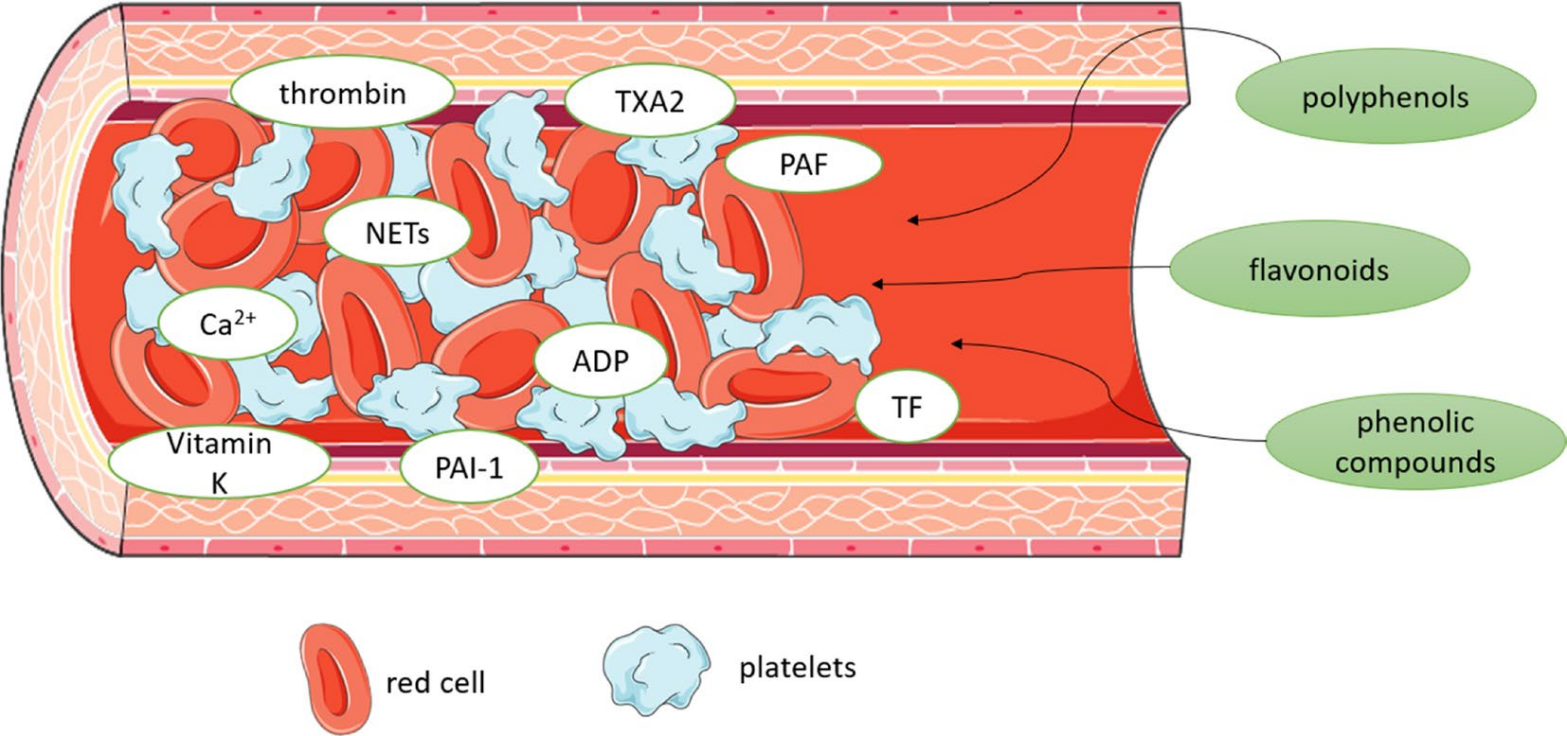
Schematic representation of thrombosis and the modulatory effects of flavonoids, polyphenols and other phenolic compounds on platelet activation, coagulation pathways and inflammatory mediators involved in clot formation. ADP: adenosine diphosphate, Ca^2+^: cytosolic calcium, NET: neutrophil extracellular traps, PAF: platelet‐activating factor, PAI‐1: plasminogen activator inhibitor‐1, TF: tissue factor, TXA2: thromboxane A2.

#### 2.4.1 Examples of Compounds With Antithrombotic Action

The use of herbal medicinal products as alternatives to synthetic antithrombotic products is gaining momentum, mainly due to their wide availability and potential cost‐effectiveness. According to the World Health Organization (WHO), 80% of the world's population depends on plants for their primary healthcare, especially in developing countries. These cost‐effective pharmaceutical compounds have significant biological activities including anticoagulant and antiplatelet properties with promising results in experimental models of thrombosis (Sawetaji and Aggarwal 2022). Thousands of flavonoids have been successfully isolated, and their number continues to grow. Scientific research confirms that the consumption of plant‐based food supplements reduces the risk of thromboembolic events, making them an effective natural treatment option (Jia et al. 2021). However, further clinical studies are required to fully establish their efficacy and safety.

##### 2.4.1.1 Tomato Aqueous Extract—Fruitflow

Tomato aqueous extract, also known as Fruitflow, is a water‐soluble, bioactive, compound‐rich tomato extract (Duttaroy 2024; O'Kennedy, Raederstorff, et al. 2017). This compound has been extensively evaluated in both preclinical and clinical studies, consistently demonstrating efficacy in reducing platelet hyperactivity. Nevertheless, these findings should be interpreted with caution, as the observed consistency does not necessarily eliminate potential limitations related to study design and population variability, which may affect the broader applicability of the results (Duttaroy 2024). Fruit juice contains various secondary metabolites derived from tomatoes, which have beneficial effects on human health (O'Kennedy, Raederstorff, et al. 2017; O'Kennedy et al. 2021).

The presence of multiple bioactive ingredients suggests that Fruitflow exerts its antiplatelet activity via a multi‐functional mechanism, with individual compounds acting at different stages of the platelet activation and aggregation cascade (O'Kennedy, Raederstorff, et al. 2017). In experimental studies, Fruitflow has been shown to inhibit platelet aggregation mediated by key agonists such as adenosine diphosphate (ADP), collagen, arachidonic acid, and thrombin (O'Kennedy et al. 2021). In addition, intake of tomato extracts was associated with reduced integrin α_IIb_β_3_ (GPIIb/IIIa) activation, modulation of interleukin production (IL‐1β, IL‐6, IL‐10, and IL‐12), reduced expression of P‐selectin on the surface of the plasma, and decreased binding to circulating tissue factor (TF) (Duttaroy 2024; O'Kennedy, Raederstorff, et al. 2017). These effects on TF binding suggest a potential effect on the coagulation process, including the production of thrombin—a mechanism critical to thrombotic events (Duttaroy 2024).

Fruitflow has also been shown to inhibit the secretion of platelet granules and to modulate the size and stability of platelet aggregates (Duttaroy 2024). In addition, it inhibits the activity of angiotensin‐converting enzymes (ACES) and promotes relaxation of the vascular endothelium (O'Kennedy, Raederstorff, et al. 2017). A clinical study demonstrated that a standard dose of Fruitflow (3 g, providing at least 65 mg of active antiplatelet compounds) exhibited antiplatelet effects comparable to those of 75 mg aspirin, including similar inhibition of thromboxane synthesis and comparable time to initial hemostatic clot formation (O'Kennedy, Crosbie, et al. 2017). Both substances have been shown to modulate the expression or activity of 26 platelet‐related proteins involved in key processes such as platelet activation, aggregation, adhesion, and blood coagulation (Duttaroy 2024).

The potential of tomato extracts in water preparations—often marketed under the name Fruitflow—to control platelet aggregation has been extensively investigated. These extracts have been shown to exert antiplatelet activity by inhibiting important signaling pathways including collagen and thrombin. The potency of these extracts is dependent on the standardized concentrations of specific bioactive metabolites, although in vitro and in vivo studies confirm their efficacy. These extracts are incorporated effectively in a wide range of food matrix and supplement formulations and offer a consistent method to investigate the cardiovascular health potential of tomato‐derived bio actives as a preventive measure (Duttaroy 2024; O'Kennedy, Raederstorff, et al. 2017).

##### 2.4.1.2 Red Yeast Rice (RYR)

RYR, also known as Beni‐koji, is a naturally fermented product obtained from the cultivation of *Monascus* species in white rice (Banach et al. 2022). It is a traditional foodstuff in several Asian countries and has been used for a long time in traditional Chinese medicine (Banach et al. 2022). The fermentation process involves *Monascus* fungi, commonly known as red koji, which produce the characteristic red pigments responsible for the deep red coloration of RYR (Fukami et al. 2021). To date, around 20 strains of *Monascus* have been identified, most of which are associated with the production of RYR (Fukami et al. 2021). In short, the production process consists of soaking the rice in water for about 7 days, then steaming the cleaned rice and inoculating it with starter cultures derived from the grape leaves of Shaoxing. Fermentation is then carried out in controlled conditions over a period of approximately 1 week (Banach et al. 2022; AFG et al. 2019). The chemical composition of RYR consists of approximately 25%–73% carbohydrates (mainly starch), 14%–31% proteins, 2%–7% water, and 1%–5% fatty acids, as well as riboflavones, isoflavones, polyketides, and pigments such as rutropinones and monascorbam (AFG et al. 2019). The fermentation results in a complex combination of bioactive compounds known as monacolins, which, together with the pigments, are the most common and biologically active ingredients of RYR (AFG et al. 2019; Zhu et al. 2023). To date, 25 pigments and 23 monacolins have been isolated from RYR (Zhu, Qi, et al. 2019). Monacolin exists in either an acid or lactone state, depending on environmental conditions, in particular pH (Fukami et al. 2021). Among these, monacolin K (MK) is the most extensively studied and potent inhibitor of cholesterol synthesis. It was first isolated from the *Monascus* strain by Professor Akira Endo in 1979 and found to be structurally identical to lovastatin (Fukami et al. 2021). Statins such as lovastatin are widely used worldwide as serum cholesterol‐lowering agents and are the first‐line treatment for cardiovascular disease associated with atherosclerosis (Fukami et al. 2021). Subsequently, other compounds related to MK were also identified in RYR (Fukami et al. 2021; AFG et al. 2019).

The principal bioactive constituent MK, which is chemically identical to Lovastatin and thus functions as a naturally occurring inhibitor of HMG‐CoA reductase, the rate‐limiting enzyme in endogenous cholesterol biosynthesis (Banach et al. 2022). The content of RYR in MK shows significant variability and lack of standardization, as there is no harmonized regulatory framework in Europe and manufacturers rarely disclose its levels (Zhu, Qi, et al. 2019). Although the European Food Safety Authority (EFSA) has recognized that a daily intake of 10 mg of MK contributes to the maintenance of normal levels, containing MK to be pharmacologically equivalent to statins subjects them to regulatory controls (Zhu, Qi, et al. 2019).

RYR has been shown to improve human endothelial function and contains statins and other biologically active ingredients that have beneficial effects on the circulatory system (AFG et al. 2019). Statins prevent cardiovascular events primarily by inhibiting HMG‐CoA reductase, resulting in reduced cholesterol synthesis and suppression of atherosclerosis‐induced atheroma formation (Fukami et al. 2021). The risk of atherosclerotic cardiovascular disease (ASCD) is reduced through these mechanisms.

However, RYR has been associated with adverse effects, mainly attributed to MK and the potential presence of citrinin, including myopathy, hepatotoxicity, and, less frequently, peripheral neuropathy, erectile dysfunction, and anaphylactic reactions (Zhu, Qi, et al. 2019). Among these, myopathy and hepatitis are the most commonly reported (Zhu, Qi, et al. 2019).

Citrinin is a mycotoxin produced by fungi such as *Monascus purpureus*, associated with nephrotoxicity and disruption of hepatic metabolic processes, raising significant safety concerns in RYR products (Zhu, Qi, et al. 2019). It has been detected in a high proportion of commercial preparations, while the EFSA has established a level of no concern for nephrotoxicity at 0.2 μg/kg body weight per day (Zhu, Qi, et al. 2019).

## 3 Antithrombotic and Antiplatelet Properties of Natural Products

### 3.1 Main Compounds With Antiplatelet Properties

Several flavonoids and phenolic compounds have been shown to have potent antiplatelet and anticoagulant properties. Pentamethyl quercetin (PMQ), a polymethyl analogue of quercetin, has been shown to inhibit the formation of blood clots and improve blood flow in mouse models when carotid artery injury is present, which highlights its potential for inhibition (Vazhappilly et al. 2019). Other bioactive flavonoids, including caffeine, galangin, apigenin, quercetin, camphorol, and ferulic acid, have been reported to inhibit platelet aggregation in a dose‐dependent fashion (Vazhappilly et al. 2019).

*Phenolic compounds* such as cyanide, epicatechin and silybin contribute to the antiplatelet activity by reducing the activity of thrombin. Other flavonoids, including genistein, apigenin, and luteolin, inhibit collagen‐ and ADP‐induced thromboxane A2 (TXA2) aggregation and inhibition of TXA2 production by binding to receptors (Mazumder et al. 2022).

*Nobiletin*, a polymethoxylated flavone extracted from citrus fruit, also has anti‐thrombotic effects by reducing the level of cytosolic calcium (Ca^2+^) in the blood and thereby inhibiting the coagulation cascade (Vazhappilly et al. 2019). Cytoplasmic Ca^2+^ overload is directly correlated to the activation of the platelets and the formation of clots. Similarly, quercetin extracted from propolis has been shown to inhibit the mobilization of Ca^2+^ in platelets, which further enhances its antiplatelet activity (Jia et al. 2021).

*Isoflavonoids* such as genistein and daidzein, flavonoids such as silybin and silyclithine, and polyphenols such as resveratrol, green tea catechins, and quercetin have been shown to prevent thrombotic disorders by different mechanisms, with polyphenols mainly targeting TF (Vazhappilly et al. 2019). In particular, resveratrol may inhibit the platelet signaling pathways by reducing P‐selectin expression, thereby inhibiting the activation of the platelets (Mazumder et al. 2022).

In addition, *coumarin‐rich* NPs have been shown to antagonize vitamin K and inhibit the coagulation cascade (Subramani and Sathiyarajeswaran 2022). The beneficial effects of flavonoids on cardiovascular diseases and thrombosis are well‐documented. For instance, Hrubeshka and colleagues investigated the flavonoid metabolites, in particular, 4‐methylcatechol and its derivatives, which have been shown to be very potent antiplatelet agents, approximately 10‐fold stronger than acetylsalicylic acid (ASA). Their mechanism is inhibition of cyclooxygenase (COX) activity, resulting in reduced production of TXA2 and reduced platelet aggregation. Four metabolites—4‐ethylcatechol, 4‐fluorocarboxylic acid, 2‐methylcatechol, 4‐methylphenol, and 3‐methylcatechol—have been identified as both safe and highly potent, but further studies are needed to confirm their clinical potential (Hrubša et al. 2022).

*Terpenes and alkaloids*. In addition to flavonoids and polyphenols, other important classes of natural substances with antithrombotic potential are alkylating agents and terpenes. Alkaloids are cyclic organic compounds with heterocyclic tertiary nitrogen atoms, with the notable exceptions of colchicine, paclitaxel, and caffeine. Based on their molecular structure, alkaloids are classified in different subclasses. Studies with medicinal plants have shown that many alkaloids have antiplatelet activity. For example, berberin was shown to inhibit platelet aggregation induced by ADP and collagen by inhibition of TXA2, and another study found that 7 of the 11 isoquinoline alkaloids significantly reduced the activity of TF. The main ingredients of essential oils are terpenes, also called isoprenoids. They are made of isoprene units, each of which contains five double bonded carbon atoms. Based on the number of isoprene units, terpenes are classified as monoterpenes, sesquiterpenes and diterpenes, each containing 2, 3, and 4 isoprene units, respectively (Mazumder et al. 2022). Research with hawthorn leaf extract has shown that nine monoterpenoids and one diterpenoid have potent inhibitory effects on platelet aggregation induced by ADP (Gao et al. 2019). The principal bioactive compounds reported to exert antiplatelet effects, along with their underlying mechanisms of action, are summarized in Table 2.

**TABLE 2:** Major compounds exhibiting antiplatelet activity and their mechanisms of action.

Compound	Chemical formula	Biological activity	References
Pentamethyl quercetin (PMQ)	C_20_H_20_O_7_	Inhibits blood clot formation and improves blood flow in mouse models with carotid artery injury, highlighting its potential as an antithrombotic agent	Vazhappilly et al. (2019)
Nobiletin	C_21_H_22_O_8_	Exhibits antithrombotic activity by reducing cytosolic calcium (Ca^2+^) levels, thereby inhibiting the coagulation cascade	Vazhappilly et al. (2019)
Quercetin (from propolis)	C_15_H_10_O_7_	Inhibits Ca^2+^ mobilization in platelets, enhancing its antiplatelet activity	Jia et al. (2021)
Resveratrol	C_14_H_12_O_3_	Inhibits platelet signaling pathways by reducing P‐selectin expression, thereby suppressing platelet activation	Mazumder et al. (2022)
4‐Methylcatechol	C_7_H_8_O_2_	Inhibits cyclooxygenase (COX) activity, resulting in reduced thromboxane A2 (TXA2) production and decreased platelet aggregation	Hrubša et al. (2022)

### 3.2 Mechanism of Antithrombotic Properties of NPs

The antithrombotic mechanisms of NPs appear to involve multiple pathways, including modulation of platelet bilayers, regulation of ROS, reduction of oxidative stress, alteration in intracellular Ca^2+^ concentrations, and inhibition of key enzymes such as phospholipase C, cAMP‐phosphodiesterase, COX, and TXA2 synthase. For instance, polyphenol epigallocatechin gallate (EGCG) has been shown to increase the levels of cAMP during collagen‐induced platelet activation and thereby to prevent platelet aggregation (Mazumder et al. 2022).

The common mechanism shared by all flavonoids appears to be the stabilization of the platelet membrane, which contributes to their general antiplatelet activity in the assays for ADP‐induced aggregation (Bojić et al. 2019). In particular, analysis of a group of 30 aglycone flavonoids showed that all tested compounds inhibit platelet aggregation at effective concentrations ranging from 0.12 mg (e.g., 3,6‐dihydroxyflavone and fusarium) to 122 mg (e.g., galangin and fusarium). Analysis of structural and activity relationships revealed that the antiplatelet activity is dependent on the configuration of the C ring, which defines the flavonoid class. In unmethylated compounds, the double bond between C2 and C3 increases activity, and the presence of the hydroxyl group at Position 6 further enhances the activity. In contrast, methylation of the A and B rings reduces antiplatelet activity (Bojić et al. 2019).

#### 3.2.1 Effect on TXA2

Flavonoids exert their antiplatelet activity in part by inhibiting the production of TXA2, a major mediator of platelet aggregation (Jia et al. 2021). In physiological conditions, TXA2 and prostacyclin (PGI2) exist in a dynamic equilibrium that regulates blood coagulation. Excess production of TXA2 disrupts this balance, leading to platelet aggregation, lipid peroxidation, and free radical formation due to impaired PGI2 clearance. PGI2 normally prevents platelet aggregation.

Flavonoids increase PGI2 production and contribute to their antiplatelet properties (Jia et al. 2021). Among flavonoids, catechins are particularly active, and compounds such as salminanolic acid A and quercetin have been shown to be potent TXA2 inhibitors (Jia et al. 2021; Bojić et al. 2019) whereas isoflavonoids tend to be less potent. It is interesting to note that polyhydroxyflavonoids can induce platelet aggregation in the presence of arachidonic acid challenge. This effect is thought to be related to their antioxidant properties, which increase the conversion of prostaglandin G2 (PGH2) to prostaglandin H2 (PGH2). PGH2 is then converted to TXA2 by TXA2 synthase, which is a potent inducer of platelet aggregation (Bojić et al. 2019).

#### 3.2.2 Effect on Plasminogen Activator Inhibitor‐1 (PAI‐1)

Another mechanism by which NPs may inhibit thrombus formation involves PAI‐1, which stabilizes the formation of blood clots by inhibiting the conversion of plasminogen to plasmin and thereby inhibits fibrinolysis. Experimental studies in mice have shown that natural compounds, including the flavonoids baicalein, heparin, and salvianolic acid B, may antagonize and reduce the activity of PAI‐1 (Ohkura and Morimoto‐Kamata 2024). Furthermore, polysaccharides of marine origin studied for their therapeutic potential have been shown to reduce the levels of PAI‐1 (Mazumder et al. 2022).

#### 3.2.3 Effect on Platelet Activating Factor (PAF)

Natural flavonoid‐rich products have been reported to inhibit the PAF (Subramani and Sathiyarajeswaran 2022a). Micronutrients commonly found in the Mediterranean diet (MD) are also associated with favorable regulation of PAF, either directly or indirectly. However, studies in this area are limited and further research is needed to clarify whether the reduction in PAF activity contributes to the protection of the MD against chronic disease (Nomikos et al. 2018). Among the few studies reported that platelet sensitivity to PAF‐ and ADP‐induced aggregation decreased after 4 weeks of the MD, while other cardiometabolic risk factors remained unchanged (Karantonis et al. 2006). Similarly, in another study, it was found that adherence to MD guidelines was associated with a decrease in plasma levels of PAF and a decrease in the activity of lyso‐PAF acetyltransferase (Lyso‐PAF AT), a key enzyme involved in the biosynthesis of PAF (Detopoulou et al. 2015). Overall, typical “healthy” components of the MD—including cereals (dietary fiber), legumes, vegetables, fish (rich in omega‐3 fatty acids), olive oil, and wine—appear to contribute to PAF regulation and may play a role in the diet's cardioprotective effects (Nomikos et al. 2018).

#### 3.2.4 Effect on Neutrophil Extracellular Traps (NETs)

NPs and bioactive substances from traditional Chinese herbal medicinal products (TCHM) have been shown to have antithrombotic effects by targeting NETs (Lei et al. 2024). NETs are structures composed of decondensed chromatin, azurophilic granules, and cytoplasmic proteins released by neutrophils (Jorch and Kubes 2017). Several constituents of NETs, such as MPO‐DNA complexes and extracellular double‐stranded DNA, are involved in thrombosis. This highlights their role in the pathophysiology of thrombotic diseases (Lei et al. 2024).

NETs are closely related to platelet aggregation. Activated platelets interact with neutrophils to induce NETosis, which is a scaffold for platelet aggregation, coagulation factors, and von Willebrand Factor (vWF) (Carestia et al. 2016). Platelets also secrete P‐selectin, TXA2, platelet factor 4 (PF4), vWF, fibronectin, and fibrinogen, which further promote the formation of NETs and enhance platelet adhesion, aggregation, and fibrin formation (Etulain et al. 2015; Stark and Massberg 2021). The release of HMGB1 from the platelets activates the TLR4 of neutrophils and produces NETs, and also activates the TLR4 and MyD88 on the platelets' surfaces, resulting in the activation of cGMP‐dependent platelet activation (Stark et al. 2016). Histones in the NETs, especially H3 and H4, promote platelet aggregation through direct interaction with fibrinogen and vWF. They contribute to prothrombin conversion to thrombin and activate platelets by facilitating Ca^2+^ entry (Mandel et al. 2022; Sharma et al. 2021; Yao et al. 2023). Additionally, they indirectly contribute to thrombogenesis by inducing endothelial injury and promoting the expression of TF, thereby triggering activation of the coagulation cascade. More specifically, NET components—including DNA, proteins, and histone H4—further enhance thrombogenesis by increasing TF activity, stimulating factors XIIa and XIa, and promoting thrombin generation (De Los Reyes‐García et al. 2019; Kim et al. 2016; Pozzi and Di Cera 2016). NETs interact with TF and vWF to enhance coagulation (Demyanets et al. 2020). In addition, NETs contribute to resistance to fibrinolysis; decondenzed DNA binds fibrin, plasmin, and elastase, and histones associated with NETs block the degradation of tPA (Gould et al. 2015; Laridan et al. 2017). Taken together, these findings highlight the critical role of NETs in the formation of blood clots and underscore the importance of the discovery of natural compounds that target NETs for antithrombotic effects. The following table (Table 3) summarizes the active substances derived from TCHM that are associated with this property.

**TABLE 3:** Natural products from Chinese herbal medicines and their antithrombotic activity against NETosis.

Natural products from Chinese Herbal Medicines	Antithrombotic therapy targeting NETS	References
Natural glucosides		
Glycyrrhizin	Inhibits HMGB1 directly by blocking the activation of its receptors, such as TLR4 and MyD88	Stark et al. (2016); Yang et al. (2015); Xue et al. (2021)
Chikusetsusaponin V	Suppresses NETosis by blocking hepatocyte‐neutrophil interaction via the HMGB1 receptor—MPO inhibition reduces chromatin decondensation and NET‐induced thrombosis	Liu et al. (2021); Thiam et al. (2020)
Forsythiaside B	– In zebrafish, reduces neutrophil/migration by lowering ROS and inflammatory factors – In rats, decreases coagulopathy via PAD4‐dependent NETosis inhibition	Gong et al. (2020); He et al. (2022)
Natural alkaloids		
Berberine	It enhances miR‐340‐5p expression thereby inhibiting HMGB1 secretion	Long et al. (2023)
Colchicine	In mice, suppress NETosis by reducing NOX2 and Ca^2+^. Clinically NETosis is linked to IL‐1β secretion	Li, Yuan, et al. (2019); Nidorf et al. (2020); Cimmino et al. (2023)
Tetramethylpyrazine also known as ligustrazine	– Inhibits ROS generation and NETosis by suppressing NOX – In zebrafish, also reduces fibrinogen and vWF expression	Liu et al. (2021)
Tetrandrine	In mice, it significantly reduced the levels of NE, PAD4, MPO, and CitH3, thereby decreasing the formation of NETs	Zhang et al. (2022)
Quinones		
Tanshinone IIA	– Suppresses NET release and promotes neutrophil apoptosis. – Improves endothelial dysfunction and inhibits HMGB1 expression/release	Zhang et al. (2017); Feng et al. (2021); Zhao et al. (2017)
Emodin	In murine arthritis, inhibits NETosis, reduces granulocyte damage, and lowewrs Cit‐H3 and PAD4	Zhu, Yuan, et al. (2019); Mei et al. (2020); Li, Miao, et al. (2019)
Terpenoids		
Andrographolide	Inhibits thrombin‐induced platelet aggregation, promotes LPS‐induced neutrophil apoptosis and reduces NETs formation	Li, Yuan, et al. (2019)
Senkyunolide I	Decreases TNF‐α, IL‐1β, IL‐6, and MPO‐DNA complex levels	Lei et al. (2024)
Tryptolide	Anti‐NETosis effects are linked to anti‐inflammatory, not oxidative stress regulation	Huang et al. (2018); Guan et al. (2021)
Dihydrotanshinone I	Stabilizes vulnerable plaques and prevents atherothrombosis by the suppressing MPO and NOX activity during NETosis	Zhao et al. (2021); Tao, Xu, Dai, et al. (2018)
Flavonoids		
Luteolin	– Inhibits collagen‐induced platelet aggregation and ROS production – Limits NETosis via the RAF1‐MEK‐1‐ERK pathway	Ye et al. (2023); Yang et al. (2018)
Quercetin	– In vitro, reduces NET release from activated neutrophils – Inhibits MPO and NE activity – Protects endothelial cells by lowering MPO and NOX	Lei et al. (2024); Pereira et al. (2024)
Phenolic acids		
Salvianolic acid B	Attenuates MPO and NOX activity, exerting antithrombotic effects via NET inhibition	Tao, Xu, Dai, et al. (2018)
Extracts		
Ginsenoside Rgx365	Reduces histone H4, MPO and NE levels associated with NETosis	Park et al. (2021)
Terpenoids of *Celastrus orbiculatus*	Reduce NOX‐dependent NETosis in PMA‐induced neutrophils, demonstrating their anticoagulant activity	Tao, Xu, and Liu (2018)
The solubilized ginger extract	Inhibits NETosis and reduces circulating NETs levels in healthy individuals	Ali et al. (2023)
Compounds		
Xuebijing injection (XBJ)	Decreases CitH3, MPO, and NE expression, suppressing GSDMD‐related genes and NET formation	Shang et al. (2022)
Traditional Chinese Herbal Medicine (TCHM) formulations	Regulation of NET‐ relatedprocesses is important, but its link to antithrombotic effects remains unclear	Zhou et al. (2023); Le‐xin et al. (2024); Yang et al. (2021)

Apart from TCHM, accumulating studies suggest that various bioactive compounds from NPs, particularly flavonoids, display pronounced anti‐NETotic activity (Hasnat et al. 2024). Τhis regulatory activity is mainly mediated through two fundamental mechanisms: (i) the direct scavenging of ROS and (ii) the inhibition of peptidyl arginine deiminase 4 (PAD4) enzymatic activity. Flavonoids represent a broad class of natural compounds with diverse biological properties (Ullah et al. 2020). A common feature shared by almost all flavonoid subclasses is their pronounced antioxidant capacity, which contributes to cellular protection against the deleterious effects of free radicals and (Zahra et al. 2024). Below, selected flavonoids and their contribution to the limitation of NETosis are presented and summarized in Table 4.

**TABLE 4:** Selected flavonoids exhibiting inhibitory activity against NETosis.

Flavonoids	Main anti‐NETosis mechanism	References
Luteolin	Inhibits NET formation mainly by suppressing the Raf1–MEK–ERK signaling pathway and reducing ERK phosphorylation, leading to decreased ROS production, neutrophil activation, and degranulation	Yang et al. (2018); Hakkim et al. (2011); Askarizadeh et al. (2025)
Epigallocatechin‐3‐gallate (EGCG)	Inhibits neutrophil elastase (NE) activity, thereby preventing histone degradation and chromatin decondensation required for NETosis	Li et al. (2022)
Naringenin	Indirectly suppresses NETosis by inhibiting NF‐κB and NOX2 signaling, reducing inflammatory activation, ROS generation, and neutrophil recruitment	Askarizadeh et al. (2025); Nouri et al. (2019)
Catechin	Attenuates NETosis primarily through antioxidant activity, inhibition of MPO and NADPH oxidase, and downregulation of PAD4 expression	Askarizadeh et al. (2025); Kirchner et al. (2010)
Quercetin	Suppresses NETosis by inhibiting neutrophil elastase release and activity and negatively regulating the P2X7R/p38 MAPK/NOX2 axis involved in ROS‐dependent NET formation	Askarizadeh et al. (2025); Tóth et al. (2017)
Taxifolin	Selectively inhibits NETosis via activation of the Nrf2 pathway and suppression of the ROS–PAD4 axis, preventing chromatin decondensation	Askarizadeh et al. (2025); Das et al. (2021)
Kaempferol	Selectively inhibits NETosis through targeted inhibition of PAD4 without impairing essential neutrophil functions, reducing histone citrullination and NET markers (dsDNA, cit‐H3)	Askarizadeh et al. (2025)
Myricetin	Regulates NETosis by directly targeting PAD4 and myeloperoxidase (MPO), reducing histone citrullination and NET release in inflammatory and autoimmune models	Askarizadeh et al. (2025); Shu et al. (2024)

Luteolin, a naturally occurring flavonoid widely distributed among plant species, is chemically defined as 3′,4′,5,7‐tetrahydroxyflavone. It has been shown to exert a significant regulatory effect on neutrophil function by limiting processes such as chemotaxis, ROS production, elastase release, and the formation of NETs (Yang et al. 2018). Beyond these effects, accumulating evidence indicates that luteolin inhibits Raf1 activity and modulates the Raf1–MEK–ERK signaling cascade in neutrophils, a pathway recognized as a critical regulator of NET formation (Hakkim et al. 2011). Furthermore, in vitro studies demonstrated that luteolin significantly attenuates ERK phosphorylation in activated human neutrophils without substantially affecting p38 MARK and JNK phosphorylation at a single dose of 50 mg/kg, it also reduced superoxide anion levels, elastase release and MPO expression (Askarizadeh et al. 2025).

Epigallocatechin‐3‐gallate (EGCG) is one of the most prominent polyphenolic constituents of green tea and is regarded as a bioactive compound of considerable scientific interest (Zhang et al. 2022). Numerous experimental studies have highlighted the ability of EGCG to inhibit the functional activity of neutrophil elastase (NE), a key enzyme involved in inflammatory responses (Li, Yuan, et al. 2019). NETosis is orchestrated by multiple molecular mediators, among which NE plays a central role. Upon neutrophil activation, NE translocates from azurophilic granules to the nucleus, where it proteolytically degrades histones, leading to chromatin relaxation. In cooperation with myeloperoxidase (MPO), NE further promotes chromatin decondensation, a critical step preceding NET release (Askarizadeh et al. 2025). EGCG, administered at 10 mg/kg daily for 5 days in vivo and at 50 mg/kg for 1 week ex vivo, resulted in reduced MPO activity (Askarizadeh et al. 2025).

Naringenin, also known as 4′,5,7‐trihydroxyflavanone, belongs to the class of polyphenolic flavonoids and is predominantly found in citrus fruits such as lemons, oranges, mandarins, and grapefruits (Lee et al. 2001). Its effect on NETosis is primarily indirect and arises from its modulatory influence on inflammatory and oxidative signaling pathways involved in neutrophil activation. Specifically, naringenin suppresses NF‐κB signaling, thereby reducing the production of pro‐inflammatory cytokines and overall neutrophil activation (Nouri et al. 2019). Naringenin, administered at 50 mg/kg for 30 days in vivo, significantly inhibited inflammatory markers TNF‐α, IL‐1β, IL‐6, and NF‐κB activation. Concurrently, it decreases oxidative burden by inhibiting NADPH oxidase 2 (NOX2), a key initiator of NETosis. In addition, naringenin downregulates the expression of the adhesion molecule ICAM‐1, limiting neutrophil recruitment and tissue accumulation, which ultimately leads to reduced NET formation (Askarizadeh et al. 2025).

Catechin modulates NETosis primarily through its potent antioxidant activity. It efficiently neutralizes reactive oxygen and nitrogen species, which are major molecular triggers of NET formation. Catechin, at concentrations of 4–100 μM in vitro, inhibited ROS production and NETosis (Askarizadeh et al. 2025). Moreover, catechin inhibits the activity of key enzymes involved in ROS generation, such as MPO and NADPH oxidase, thereby reducing the oxidative environment required for NETosis (Kirchner et al. 2010). Beyond its direct enzymatic effects, catechin also appears to regulate NETosis at an epigenetic level by downregulating PAD4 expression in activated neutrophils (Askarizadeh et al. 2025). Through these combined mechanisms, catechin disrupts the self‐amplifying inflammatory loop associated with excessive NET production, rendering it, particularly, relevant in autoimmune and chronic inflammatory disorders (Askarizadeh et al. 2025).

Quercetin is one of the most commonly consumed dietary flavonoids and is widely present in plant‐based foods such as onions, apples, and grapes (Di Petrillo et al. 2022). Experimental studies have demonstrated that quercetin reduces both the release and catalytic activity of BE, a crucial enzyme for histone degradation during NETosis. In addition, quercetin negatively regulates the P2X7R/p38 MAPK/NOX2 signaling axis, which is closely associated with ROS‐dependent NET formation. Quercetin, at a concentration of 100 μM for 30 min in vitro, inhibited NETosis (Askarizadeh et al. 2025). Its anti‐inflammatory properties are further reflected in the inhibition of neutrophil accumulation and degranulation, as well as in the reduction of MPO activity, as shown in murine models of intestinal ischemia–reperfusion injury (Tóth et al. 2017). Notably, quercetin also exhibits therapeutic potential in cardiovascular diseases, including myocardial infarction. During the early phases of reperfusion, polymorphonuclear neutrophils infiltrate myocardial tissue and contribute to infarct expansion through the release of ROS and proteolytic enzymes. The ability of quercetin to simultaneously inhibit protein kinase C and the proteasome—two key regulators of NETosis—renders it particularly effective in this pathological context (Askarizadeh et al. 2025).

Taxifolin is a polyphenolic compound characterized by strong antioxidant and anti‐inflammatory properties; however, its most remarkable feature is its capacity to selectively inhibit NETosis through mechanisms mediated by activation of the nuclear factor erythroid 2–related factor 2 (Nrf2) pathway (Das et al. 2021). Nrf2 serves as a central regulator of cellular antioxidant defenses by inducing the expression of cytoprotective proteins and limiting neutrophil activation (Zhu et al. 2023). Activation of Nrf2 by taxifolin effectively targets the ROS–PAD4 axis, resulting in inhibition of chromatin decondensation, a pivotal step in NET formation. Taxifolin, at a concentration of 0.1–1 mg/mL in vitro, inhibited ROS production and reduced IL‐1β, IL‐6, IFN‐γ levels, as well as NETosis. This highly specific mechanism places taxifolin among the few polyphenols with such targeted anti‐NETotic activity. Moreover, taxifolin has been shown to attenuate NET‐driven venous thrombosis by reducing key NETosis mediators, including citrullinated histone H3 and NE activity (Askarizadeh et al. 2025).

Kaempferol is a widely distributed dietary flavonoid found in foods such as apples, strawberries, tomatoes, broccoli, green tea, and *Ginkgo biloba* leaves (Kim and Choi 2013). Recent evidence highlights its unique ability to selectively suppress NETosis through direct inhibition of PAD4, without impairing the essential antimicrobial functions of neutrophils. By blocking histone citrullination and subsequent chromatin decondensation, kaempferol interferes with critical steps required for NET release. Both in vitro and in vivo studies have demonstrated significant reductions in NETosis markers, including double‐stranded DNA and citrullinated histone H3. Kaempferol, at a concentration of 25 μmol/L administered twice daily for 24 h in vitro, reduced cit‐H3 levels. Importantly, kaempferol has also been implicated in the inhibition of cancer metastasis; in breast cancer models, it significantly reduced metastatic spread through suppression of NET formation (Askarizadeh et al. 2025).

Myricetin is predominantly found in *Pterocarya rhoifolia* and is distinguished by its multifaceted regulatory action on NETosis. Unlike many other flavonoids, myricetin directly targets two critical mediators of NET formation: MPO and PAD4, which are infrequently targeted simultaneously by natural compounds (Shu et al. 2024). Myricetin reduces both the expression and enzymatic activity of these mediators in neutrophils, thereby limiting histone citrullination and NET release. Myricetin, administered at 5 mg/kg daily for 6 weeks in vitro, significantly reduced ROS levels. Experimental evidence from in vitro and in vivo models of inflammation and rheumatoid arthritis demonstrates a marked reduction in NETosis markers, supporting the therapeutic potential of myricetin in NET‐dependent autoimmune diseases (Askarizadeh et al. 2025).

It is important to note that not all of the aforementioned compounds act directly on the process of NETosis, certain compounds, such as luteolin, EGCG, catechin, quercetin, taxifolin, kaempferol, and myricetin, directly target key molecular mechanisms involved in Net formation (e.g., PAD4, MPO, NE and critical signaling pathways), whereas naringenin primarily exerts indirect effects by reducing inflammation and oxidative stress, which are major stimuli for neutrophil activation and NET formation.

## 4 Antithrombotic and Cardioprotective Effects of Natural Products

### 4.1 Evidence From Clinical Outcomes (Human Studies)

NPs have been of interest because of their potential anti‐thrombotic effects and their role in the prevention of cardiovascular disease (Vazhappilly et al. 2019). Epidemiological studies have shown that a higher intake of flavonoids is associated with a reduction of almost 18% in cardiovascular mortality (Ullah et al. 2020). However, these data derive from association studies and do not establish a causal relationship. While in human studies, the consumption of flavanol‐rich cocoa (~821 mg/day, orally) for 5 days has been shown to improve vascular function markers (Fisher et al. 2003).

Understanding the contribution of natural compounds to reducing cardiovascular events is of considerable importance, given that conventional therapies, such as aspirin, clopidogrel, and newer anticoagulants, although effective, may be associated with bleeding or ischemic complications (Olas 2022).

### 4.2 Evidence From Biomarkers (Surrogate Endpoints in Humans)

Many human studies have focused on intermediate markers of cardiovascular risk. Polyphenols appear to have protective effects by improving endothelial function and inhibiting the oxidation of LDL, a key factor in atherosclerosis (Mutha et al. 2021). Proteins from soy and bioactive compounds from green tea have been associated with reduced LDL levels and increased NO‐dependent vasodilation (Mutha et al. 2021). Supplementation with oral resveratrol (100 mg every 3 months) has been shown to improve endothelial function and reduce atherosclerosis (Mutha et al. 2021).

In addition, proanthocyanidins, isoflavones, and anthocyanins improve vascular health by reducing the stiffness of the arteries, further reducing the risk of cardiovascular disease (Ullah et al. 2020). Polyphenols from wine or grape juice reduce platelet aggregation and bleeding time (Mutha et al. 2021). However, these markers represent indirect outcomes and do not replace clinical endpoints.

### 4.3 Mechanistic and Experimental Evidence

The pathogenesis of myocardial infarction and ischaemic stroke is thought to be initiated by activation and accumulation of platelets (Vazhappilly et al. 2019). In vitro and in vivo studies have shown that catechins and proanthocyanidins provide vascular protection, while flavonoids present in red wine, tea, fruit, and vegetables have anti‐thrombotic effects (Vazhappilly et al. 2019). Quercetin, which is found in onions, inhibits the formation of atherosclerotic plaques and the activity of metalloproteinase I, thereby reducing the risk of coronary heart disease (Mutha et al. 2021).

Phenolic compounds with strong antioxidant activity, such as ascorbate and vitamin C, are considered to be potential cardioprotective agents as they regulate the reactive oxygen and nitrogen species (ROS and RNS) and thereby reduce the hyperactivation of the platelets and the formation of clots. Polyphenolic antioxidants target the hydrogen peroxide released by platelet aggregation interactions, which in turn cause TXA2 formation and platelet aggregation (Mazumder et al. 2022). Although the anti‐stroke effects of flavonoids are not fully understood, there is evidence that anthocyanins may modulate blood pressure by modulating NO from endothelial cells (Vazhappilly et al. 2019).

## 5 Nutrition‐Epigenetics and Cardiovascular Disease

Diet has been shown to affect the epigenome via micronutrients and bioactive compounds, many of which are derived from NPs, altering health and modifying the risk of cardiovascular disease (Kalea et al. 2018; Ghazi et al. 2020). Although the effect of nutritional epigenetics on thrombotic processes is not fully understood, current evidence primarily links epigenetic mechanisms to overall cardiovascular disease rather than thrombosis specifically. Most findings concern the regulation of broader cardiovascular risk factors and pathways, and direct links between nutritional epigenetics and thrombosis per se remain insufficiently clarified.

Epigenetic mechanisms involved in cardiovascular disease include DNA methylation, posttranslational histone modification, and noncoding RNA regulation (Kalea et al. 2018). By altering the activity of DNA methyltransferases (DNMTs) and histone deacetylases (HDACs), compounds such as resveratrol and curcumin have cardioprotective effects that are less toxic and have pleiotropic benefits compared to synthetic drugs. Pharmacotherapeutic efficacy in cardiovascular disease is still poorly studied despite its potential, underlining the need for more mechanistic research, improved delivery methods, clinical validation, and tailor‐made treatment strategies (Martínez‐Iglesias et al. 2025).

However, it should be emphasized that most of the studies in this area were performed in vitro, and therefore further research is needed to confirm these findings in vivo (Ghazi et al. 2020). Epigenetics refers to reversible and inheritable changes in the function of genes without altering the DNA sequence of the individual genes (Kalea et al. 2018).

### 5.1 DNA Methylation

DNA methylation is a key epigenetic alteration that regulates gene expression and is clearly associated with cardiovascular disease (Ghazi et al. 2020; Wallace et al. 2018). Alterations in the methylation patterns of DNA may impair vascular function and may promote the formation of atherosclerotic plaques (Kalea et al. 2018). Dietary micronutrients affect DNA methylation via the metabolism of one‐carbon (1C), which provides methyl groups for DNA synthesis, amino acid synthesis, phospholipid synthesis, and protein synthesis (Ghazi et al. 2020). This process involves bioactive compounds such as resveratrol, curcumin, genistein, sulforaphane, phenethyl isothiocyanate, and EGCG (Ghazi et al. 2020; Shankar et al. 2013). Among these, folate (vitamin B9) is the most extensively studied nutrient directly involved in the methylation of DNA (Ghazi et al. 2020).

### 5.2 Histone Modifications

Histone modifications are also critical in chromatin remodeling and regulation of genes (Shankar et al. 2013). Dietary compounds such as EGCG, genistein, curcumin, and resveratrol affect the acetylation of histones by inhibiting the activity of histone acetyltransferase (Shankar et al. 2013). In addition, saffron ( *Crocus sativus* ) has been shown to interact with the binding of histone H1 via its carotenoid components, suggesting that it may play a role in the regulation of epigenetics (Rashid et al. 2022).

### 5.3 MicroRNAs (mRNAs)

mRNAs are small single‐stranded RNA sequences (Kura et al. 2019), whose physiological function is to inhibit genes by destabilizing or preventing the translation of mRNA (Zempleni et al. 2015). They play a key role in gene silencing and therefore seem to have a major effect on the expression of proteins throughout the body and play a major role in cardiovascular pathways (Kura et al. 2019), with 150–200 miRNAs detected in the cardiovascular system (Collado et al. 2021) and also associated with cardiovascular disease (Kura et al. 2019). Modern studies combine the actions of these molecules with diet‐induced cardioprotection. This is demonstrated by changes in the expression of many endogenous miRNAs, as well as by exogenous miRNAs (exog‐miRNAs) present in the diet with the potential for entering the blood and tissues (Kura et al. 2019; Collado et al. 2021), such as lycopene and dietary fibers (Collado et al. 2021). Regarding the properties of exog‐miRNAs, recent studies have suggested that these miRNAs present in food, entering the body via the gastrointestinal tract may also regulate gene expression (Kura et al. 2019). They are transported in exosomes (Kura et al. 2019).

Main sources of exog‐miRNAs are plant‐derived foods and also cow's milk. Although structural changes and loss of stability due to food processing can be seen in miRNAs (Collado et al. 2021), experiments with miRNAs from milk have shown that they can modify gene expression (Cui et al. 2017).

The miRNA expression is modulated by various natural, biologically active substances, commonly known as natural miRNA modifiers, which have cardioprotective effects. Numerous studies have shown that resveratrol reduces miR‐34a and miR‐155, which restores the activity of SIRT1 and reduces the severity of myocardial hypertrophy, fibrosis, and ischaemic reperfusion injury (Boshra 2020). Flavonoids such as kaempferol and quercetin have been shown to exert similar effects on miRNAs mediating cardioprotective pathways. These compounds regulate miR‐21 and miR‐155, which reduce oxidative stress, inflammation, proliferation of vascular smooth muscle cells, and myocardial damage (Deng et al. 2020). Other natural substances that modulate important miRNAs involved in atherosclerosis, angiogenesis, hypertrophy, fibrosis, and heart injury include genistein, ciclosporin, pterostimuline, galic acid, apigenin, luteolin, ampelopyrin, puerin, and geniposide. All these support the potential of NPs as epigenetic modulators for preventing and treating cardiovascular disease and highlight the central role of miRNAs as a key mechanism for the cardiovascular benefit of NPs.

## 6 Interaction of Natural Products With Antithrombotic Drugs

Drug–drug interactions (DDIs) are another topic that receives attention in both clinical practice and research. Recently, we have concentrated on DDIs between antineoplastic and anticoagulant medications (Yerolatsite et al. 2025). Attention also is being paid to the interaction between plant‐based medications and antithrombotic drugs. This draws attention to significant knowledge gaps and supports updates in this rapidly developing field. Through observational therapy and reverse pharmacology, herbs serve as a source of new compounds and are crucial in the discovery of NPs. 80% of people worldwide rely on herbal remedies for their health, according to the WHO (Welz et al. 2018). A significant component of complementary and alternative medicine, herbal remedies are frequently preferred due to cultural customs, familial traditions, and favorable past experiences. However, their widespread use emphasizes the need for caution and close observation and raises the possibility of interactions with currently available medications (Subramani and Sathiyarajeswaran 2022). According to numerous studies, certain NPs and herbal extracts can be added to antithrombotic medicinal products to increase their safety and therapeutic efficacy. Although some substances, such as ginsenosides, may exacerbate thrombosis in certain situations, others, such as EGCG, have been shown to have synergistic effects with warfarin, reducing the risk of deep vein thrombosis. Comprehensive preclinical research is required to ensure safety due to the potential risks associated with concomitant use. To successfully and safely integrate NPs into antithrombotic therapy, a deeper understanding of their therapeutic properties is needed, including structural and functional relationships, molecular targets, drug interactions, and pharmacokinetics (Li et al. 2022).

Herbal medicinal products work on modern pharmacological principles, that is, herbal–drug interactions are based on the same mechanisms of pharmacokinetics and pharmacodynamics as drug interactions (Izzo et al. 2005).

### 6.1 Pharmacokinetic Interactions

Herbal medicinal products may affect absorption, metabolism, and excretion, whereas no interactions between herbal medicinal products and drug delivery mechanisms have been reported (Izzo et al. 2005).

Warfarin is an oral anticoagulant widely used to prevent thromboembolic events in patients with a variety of conditions, including severe conditions (Tan and Lee 2021). Due to its frequent use, warfarin has been extensively investigated for interactions with NPs. The active substance is a racemic mixture of R‐ and S‐enantiomers, which are predominantly metabolized by hepatic cytochrome P450 (CYP) isoenzymes, in particular, CYP2C9, CYP2C19, CYP2C8, CYP1A2, and CYP3A4 (Tan and Lee 2021) (Table 5).

**TABLE 5:** Pharmacokinetic interaction of natural substances/feeds with warfarin.

Plant	Action	References
*Echinacea* (*purpurea* and *angustifolia*)	CYP3A4 inhibition	Di Minno et al. (2017)
Grapefruit (furanocoumarins)	CYP3A4 inhibition	Guo and Yamazoe (2004)
Green tea	Reduction of PT‐INR	Chan et al. (2011)
Chamomile ( *Matricaria chamomilla* )	In vitro inhibition of CYP1A2 and, to a lesser extent, 3A4 and 2C9	Ganzera et al. (2006)
Soybean or soy milk Soy milk	In vitro soy extracts inhibit CYP3A4 and CYP2C9	Cambria‐Kiely (2002)
Mango	Contains high concentrations of retinol, a known inhibitor of CYP2C19	Monterrey‐Rodríguez et al. (2002)
Ginseng	No effect on warfarin pharmacokinetics	Jiang et al. (2004)
Cranberry ( *Vaccinium myrtillus* )	Cranberry juice alters the activity of CYP2C9 and 3A4	Mellen et al. (2010)
Garlic (Garlic)	Inhibition of CYP2C9, 2C19, 3A4	Di Minno et al. (2017)
*Ginkgo biloba*	CYP2C9 inhibition	Von Moltke et al. (2004)
St. John's wort ( *Hypericum perforatum* )	Attacks CYP1A2, 2C9, and 3A4	Chen et al. (2004); Sprouse and Van Breemen (2016); Dostalek et al. (2011)

Studies investigating the interaction between warfarin and NPs have shown that most of the interactions are mediated by CYP2C9, CYP1A2, CYP2C19, and CYP3A4 (Tan and Lee 2021; Di Minno et al. 2017). Although some reports suggest that CYP2C9 effects may not be clinically relevant (Di Minno et al. 2017), general inhibition or induction of these enzymes may significantly alter the pharmacological activity of warfarin and the PT‐INR, potentially leading to life‐threatening or fatal adverse reactions (Table 6).

**TABLE 6:** Pharmacodynamic interaction of natural substances/feeds with warfarin.

Physical substance	Action	References
Green and leafy vegetables and certain vegetable oils	They contain a significant amount of vitamin K and excessive use of them may result in a decrease in PT‐INR	Di Minno et al. (2017)
Ginkgolides, the main chemical constituents of *Ginkgo biloba*	Does not alter warfarin pharmacodynamics, but regular PT‐INR monitoring is recommended	Jiang et al. (2005); Ge et al. (2014)
Ginsenosides, the major active components of ginseng	Inhibition of CYP1A2	Di Minno et al. (2017)

Echinacea, a widely used herbal medical product with immunomodulatory properties but no established antithrombotic activity, has been investigated for potential pharmacokinetic interaction with warfarin (Mao et al. 2019; Sharifi‐Rad et al. 2020; Golovinskaia and Wang 2021; Ahmadi, Kariman, Mousavi and Rengel, 2024). Pharmacokinetic studies involving *E. purpurea* and *E. angustifolia* suggest that Echinacea may increase the apparent clearance of (S)‐warfarin without significantly affecting its pharmacodynamic response, while no major effects were observed on the (R)‐enantiomer (Golovinskaia and Wang 2021; Abdul et al. 2010; Burlou‐Nagy et al. 2022). Although Echinacea has not been systematically associated with antithrombotic effects, these findings support its consideration as an example of a clinically relevant herb‐drug interaction mediated through pharmacokinetic mechanisms. Further experimental and clinical studies are warranted to clarify its role in thrombosis‐related pathways and potential implications for anticoagulant therapy.

NPs may affect warfarin by altering its pharmacokinetics (Table 5), affecting absorption, distribution, and metabolism, or by pharmacodynamic mechanisms, altering the effects of the drug when it is combined with other herbs or supplements (Tan and Lee 2021). In particular, modulation of the metabolism of the S‐enantiomer may alter plasma concentrations of warfarin. Another common mechanism is interference with the gastrointestinal absorption of warfarin and vitamin K (Tan and Lee 2021).

Beyond warfarin, herb‐DOAC interactions may arise through modulation of P‐glycoprotein and CYP3A4 pathways. St. John's Wort, a potent inducer of these systems, may reduce systemic exposure to dabigatran and rivaroxaban; concomitant use is not recommended (Nutescu et al. 2011).

Emerging evidence suggests a potential interaction between *Salvia miltiorrhiza* (Danshen) and rivaroxaban through CYP3A/CYP2J inhibition, although further clinical validation is needed (Wang et al. 2022).

### 6.2 Pharmacodynamic Interactions

In addition, these interactions may be additive or synergistic, and herbal preparations may enhance the effect of a traditional medicinal product or have an antagonistic effect (Izzo et al. 2005).

The antithrombotic effect of warfarin is due to the inhibition of coagulation factors dependent on vitamin K, II, VII, IX, and X (Tan and Lee 2021).

NPs may affect warfarin by altering its pharmacokinetics (Table 5), affecting absorption, distribution, and metabolism, or by pharmacodynamic mechanisms, altering the effects of the drug when it is combined with other herbs or supplements (Tan and Lee 2021).

Most foods can be safely consumed during treatment with warfarin; however, reports of adverse reactions should not be disregarded (Tan and Lee 2021). Several commonly reported herbs were listed, along with their therapeutic indications and interactions with conventional antithrombotic agents, highlighting an increased bleeding risk (Subramani and Sathiyarajeswaran 2022). Reports of adverse hemorrhagic events have been noted due to interactions between aspirin, clopidogrel, and warfarin (Subramani and Sathiyarajeswaran 2022).

Clinically relevant high‐risk combinations include anticoagulants with St. John's Wort, which may reduce anticoagulant activity, and ginger, which may increase bleeding risk through additive pharmacodynamic effects (Talasaz et al. 2025).

Clinically relevant herb‐drug interactions may also involve DOACs through pharmacokinetic mechanisms (CYP3A4/P‐gp), as with St. John's Wort, and through pharmacodynamic interactions, as ginkgo, garlic, ginger, and turmeric may increase bleeding risk. These combinations represent practical high‐risk combinations warranting avoidance or close monitoring (Grześk et al. 2021).

Therefore, careful patient education, a balanced diet, monitoring, and caution are necessary (Tan and Lee 2021; Di Minno et al. 2017). Given the widespread use of conventional medicines, patient safety concerns, and the significant economic burden on health systems, the need to explore new pharmacological pathways for drug discovery is underscored by the knowledge of herbal drug interactions and their complications.

## 7 Conclusions

Based on the evidence reviewed, the antithrombotic activity of bioactive substances present in NPs, such as polyphenols and flavonoids, was highlighted, underlining their important role in cardiovascular disease prevention and treatment. However, the effects of regular intake of these naturally bioactive substances on antithrombotic therapy remain an area that requires further clinical investigation, as potential interactions with anticoagulant agents are not yet fully understood. Nutritional epigenetics offers a new perspective on personalized medicine by examining how NPs modulate the expression of genes involved in cardiovascular health. Moreover, artificial intelligence (AI) offers a promising approach to the targeted detection of new natural antithrombotic agents. Overall, the multidimensional research on NPs and their bioactive ingredients shows that they can serve as powerful complementary or alternative strategies to reduce thrombotic risk and manage cardiovascular disease. However, further studies are required to elucidate the underlying mechanisms of action, establish safety profiles, and support integration into clinical protocols, with the ultimate goal of improving public health and quality of life.

## 8 Future Directions

In recent years, AI has progressed rapidly. AI is a wide area that includes the development of systems capable of performing tasks that normally require human intelligence. Its use in the area of NPs remains limited, mainly because the available data are multi‐dimensional, inconsistent, nonstandardized, and scattered across multiple repositories (Meijer et al. 2025). However, AI is facilitating the design and synthesis of complex NPs by creating synthetic pathways for complex molecules (Mikulak‐Klucznik et al. 2020). Research on NPs covers a variety of data types, including genomic, proteomic, metabolic, spectroscopic, and biochemical data. However, these datasets are often fragmented and difficult to integrate, which delays the discovery of new compounds (Meijer et al. 2025). In this context, AI and machine learning (ML) are valuable tools for predicting molecular properties and creating new chemical structures (Meijer et al. 2025). The development of AI models capable of effectively matching and integrating all these disparate data remains a major challenge (Meijer et al. 2025).

One proposed solution is to develop a knowledge graph on NPs that integrates chemical structures, metabolomics, genomic data, bioactivity, and other relevant information. It can be described as a comprehensive information network in which all data are linked, enabling AI systems to better understand relationships and to make more accurate predictions (Delmas et al. 2021). Creating such a graph could increase the ability of AI to mimic the thinking of NP scientists, speeding up research and making new discoveries (Gaudry et al. 2024). Protein structure prediction systems such as CASP (computer‐aided synthesis planning) may further improve the design and synthesis of new compounds. These systems allow researchers to test different chemical structures quickly, identify optimum candidates, and account for multiple constraints in manufacturing, making it easier to produce complex NPs efficiently (Levin et al. 2022). In summary, AI can provide mechanistic insights into how NPs influence thrombosis and cardiovascular diseases, paving the way for more comprehensive computational approaches that link molecular mechanisms with clinical outcomes.

AI is emerging as a powerful tool for understanding and managing complex biomedical processes directly related to thrombosis, including the role of NETs in thrombus formation. Through the analysis of extensive clinical, genetic, and multiomics data, ML algorithms contribute to the identification of risk patterns for thromboembolic events (Mishra and Ashraf 2020), while also enabling the investigation of molecular mechanisms linking NETs to coagulation activation and inflammatory responses, highlighting them as potential therapeutic targets and biomarkers (Sheng and Cui 2024; Han et al. 2024) (Table 7).

**TABLE 7:** Applications of artificial intelligence (AI) in thrombosis, NETs, and pharmacological interactions.

Data type	AI applications	Associated biological targets/processes	Clinical relevance/outcomes	References
Clinical data	Identification of risk patterns for thromboembolic events	Thrombosis, inflammatory response	Prediction of thromboembolic risk	Nouri et al. (2019)
Genetic data	Analysis of molecular mechanisms	Link between NETs, coagulation activation, and inflammation	Identification of NETs as biomarkers	Kirchner et al. (2010); Tóth et al. (2017)
Multiomics data	Investigation of large‐scale biological processes	Role of NETs in thrombogenesis	Identification of therapeutic targets	Kirchner et al. (2010); Tóth et al. (2017)
Chemical structure data	Prediction of drug–drug and drug‐natural product interactions	Molecular‐level interactions	Assessment of toxicity risk	Das et al. (2021); Shu et al. (2024); Lee et al. (2001)
Molecular pathway data	Understanding mechanisms of interactions	Association of drugs with biological pathways	Prediction of reduced drug efficacy	Das et al. (2021); Shu et al. (2024); Lee et al. (2001)
Pharmacokinetic data (absorption, metabolism, clearance)	Prediction of drug behavior in the body	Pharmacokinetic interactions	Prediction of response to antithrombotic therapies	Das et al. (2021); Shu et al. (2024); Lee et al. (2001)

Within the same framework, AI facilitates the prediction and understanding of interactions both among drugs and between drugs and NPs, through the processing of data related to chemical structures, molecular pathways and pharmacokinetic parameters such as absorption, metabolism and clearance. In this way, it becomes possible to identify interaction mechanisms, assess risks of toxicity or reduced efficacy and predict responses to antithrombotic therapies (Zhang et al. 2024; Obrezanova 2023; Spanakis et al. 2025).

Overall, the application of artificial intelligence strengthens the link between molecular mechanisms and clinical outcomes, supporting evidence‐based decision‐making and design of safer and more personalized therapeutic strategies.

## Author Contributions

**Maria Nektaria Magkoulia:** writing – original draft, investigation, writing – review and editing. **Despoina Pantazi:** writing – original draft, writing – review and editing, supervision, investigation, methodology.

## Funding

The authors have nothing to report.

## Disclosure

We confirm that no AI‐based tools were used in the preparation, writing, or revision of this manuscript. All content present herein has been developed and reviewed solely by the authors.

## Ethics Statement

The authors have nothing to report.

## Conflicts of Interest

The authors declare no conflicts of interest.

## Data Availability

The data that support the findings of this study are available from the corresponding author upon reasonable request.
